# Artificial intelligence in pharmacy practice: pharmacists’ perceptions and concerns toward implementation

**DOI:** 10.3389/fdgth.2026.1797213

**Published:** 2026-06-18

**Authors:** Amira S. A. Said, Mohammad M. Al-Ahmad, Sawsan Shanableh, Muaed Alomar

**Affiliations:** 1 Department of Clinical Pharmacy, College of Pharmacy, Al Ain University, Al Ain, Abu Dhabi, United Arab Emirates; 2 Department of Clinical Sciences, College of Pharmacy and Health Sciences, Ajman University, Ajman, United Arab Emirates

**Keywords:** artificial intelligence, concerns, perceived benefits, pharmacist, pharmacy practice

## Abstract

**Background:**

Integrating artificial intelligence (AI) has the potential to transform healthcare. AI can enhance medication management, patient outcomes, and streamline pharmacy operations. However, addressing AI limitations and pharmacists' concerns is essential to fully grasp its real future potential in healthcare and its impact on the workforce.

**Methods:**

A cross-sectional study was conducted using a previously developed pilot-tested online questionnaire with demonstrated content validity and reliability among licensed pharmacists in the UAE. The questionnaire assessed participants' demographics, perceived benefits (9 items), and concerns/barriers (18 items) regarding AI implementation in pharmacy practice. Responses were recorded using a 5-point Likert scale. Perception scores were analyzed using descriptive statistics (mean ± standard deviation) to reflect the distribution of responses. Internal consistency was high (Cronbach's *α*: benefits, 0.88; concerns/barriers, 0.90). Associations between participants' demographics and perception scores were analyzed using appropriate statistical tests, with significance set at *p* < 0.05.

**Results:**

A total of 400 participants were invited to participate in the study, of whom 340 returned completed questionnaires (response rate: 85%). Overall, participants reported positive perceptions of AI, particularly in relation to multitasking and rapid data analysis (4.1 ± 0.9), improved service quality (3.9 ± 1.0), and enhanced patient follow-up (3.8 ± 1.1). However, perceptions of AI's clinical impact were more moderate, including improved patient outcomes (3.5 ± 1.1), reduced medication errors (3.3 ± 1.2), and reduced healthcare costs (3.1 ± 1.2). Major concerns included cybersecurity risks (4.2 ± 0.8), data privacy issues (4.1 ± 0.9), and potential job displacement (3.9 ± 1.1).

**Conclusion:**

In this study, AI was perceived as a promising advancement in pharmacy practice, particularly in enhancing operational efficiency, multitasking, and patient follow-up. However, significant concerns were identified, including data privacy, cybersecurity, job displacement, and the potential loss of the human element in patient care. Addressing these concerns requires ensuring that AI complements, rather than replaces, pharmacists’ roles, supported by updated education, targeted training, and clear regulatory frameworks.

## Background

Artificial intelligence has recently emerged as a revolutionary development in all fields of science, and healthcare is no exception. In fact, the increasing integration of AI into healthcare systems has improved efficiency, accuracy, and clinical outcomes (1). The recent shift in pharmacy practice from a product-oriented model to a more patient-centred approach has expanded pharmacists' responsibilities. Pharmacists are now more involved in clinical services, including medication reviews, therapeutic optimization, and individualized patient care, which require efficient data processing and informed clinical decision-making (2).

This development necessitates the inclusion of AI in advanced pharmacy practice as a tool to support and improve different aspects of patient care (3, 4). The ability of AI to simulate intelligent human behaviour in healthcare, such as data analysis, disease prediction, and personalised therapeutic recommendations, is highly desirable as it can automate repetitive tasks, reduce human error, and provide high-quality patient care (5–7). All these advancements can potentially improve medication safety, patient adherence, and overall pharmacy practice outcomes (8, 9).

Although the integration of AI holds great promise for advancing pharmacy practice, it also presents several barriers and concerns that must be carefully considered before its effective implementation (10). These include issues related to data privacy, cybersecurity, high implementation costs, technical challenges, and concerns regarding the potential impact on pharmacists' professional roles and patient interaction (11).

Several regional studies have explored pharmacists' perceptions of artificial intelligence (AI), particularly in Jordan and Saudi Arabia, reporting generally positive attitudes toward its adoption alongside concerns related to cost, training, and trust in AI-driven decisions (12, 13). However, there is a lack of research assessing pharmacists' perceptions of AI in the United Arab Emirates. Therefore, further investigation is warranted to evaluate pharmacists' perceptions, concerns, and barriers towards AI implementation within the UAE healthcare systems.

This study aimed to assess pharmacists' perceptions of artificial intelligence (AI), including perceived benefits, willingness to adopt AI, and associated concerns and barriers to its implementation in pharmacy practice among pharmacists in the United Arab Emirates.

## Method

### Study design, population, and setting

This was an observational cross-sectional study, utilizing a survey that was adapted from previously published studies (3, 4, 9, 14–16) to assess UAE Pharmacists' perceptions of benefits, concerns, and barriers regarding AI implementation in pharmacy Practice.

The study was conducted between 1 January 2024 and 31 May 2024 in accordance with the guidelines provided by the Helsinki Declaration of 1964 (revised 2013). Ethical approval was obtained from the Al Ain University Research Ethics Committee (REC) (Approval No: REC/2024/014) prior to data collection. Informed consent was obtained from all participants. The sample size was calculated using the Raosoft® online calculator, with a margin of error of 5%, a confidence level of 95%, and a response distribution of 50%, yielding a minimum required sample size of 323 participants. A total of 400 pharmacists were invited to participate in the study; however, 340 completed questionnaires were returned and included in the final analysis, resulting in a response rate of 85%.

The survey was distributed to a convenience sample of pharmacists working in different UAE hospitals or community pharmacies. The questionnaire was distributed by the research team using both online (Google Forms) and in-person (face-to-face) approaches, targeting pharmacists based on their accessibility and availability.

Convenience sampling was used in this study for its simplicity, speed, and cost-effectiveness, involving selecting participants based on their easy accessibility and availability to the researcher. Participants who expressed interest in participating were provided with a link to view the study's instructions and to provide their electronic consent before proceeding to the survey. To minimize duplicate responses, the survey was limited to one submission per participant, and responses were screened to exclude any potential duplicates. It was emphasized that participation was voluntary and all data were kept confidential and used only for research purposes.

### Questionnaire development

Anonymous data were collected through an electronic questionnaire that was distributed via Google Forms or face-to-face. Written informed consent was obtained where participation was voluntary, and all collected data were de-identified prior to analysis to maintain participant confidentiality and privacy.

The questionnaire had three sections; the first section (7 questions) collected demographic details (gender and age) and other data (qualification, nationality, years of experience, and area of practice). The second section (9 questions) and the third section (18 questions) investigated participants' perceived benefits and concerns/barriers regarding AI implementation in pharmacy practice, respectively. Participants were asked to respond to each question in section two, using a 5-point Likert scale (1 = strongly disagree; 2 = disagree; 3 = neutral; 4 = agree; and 5 = strongly agree). Questionnaire completion was estimated to take approximately 10 min.

The questionnaire was assessed for content validity by 5 senior pharmacists (>10 years' experience) in pharmacy practice, and several modifications were made accordingly. A pilot study was conducted with 10 pharmacists to test the questionnaire clarity, comprehension, and relevance, and the questionnaire was amended as required. The pilot study sample pharmacists were later excluded from the final study sample. The internal reliability of individual subscales of the questionnaire was assessed using Cronbach's alpha. The internal consistency values for the benefits and concerns/barriers sections were 0.88 and 0.9, respectively.

### Statistical analysis

The data were analyzed using SPSS statistical software package, version 26 (SPSS Inc., Armonk, New York, United States). Descriptive statistics were used to summarize participant characteristics and responses. Categorical variables were presented as frequencies and percentages. Responses to Likert-scale items were analyzed using mean scores and standard deviations (SD). Composite scores for perceived benefits and concerns/barriers were calculated by summing the respective item responses for each domain. These composite scores were treated as continuous variables, with higher scores indicating more positive perceptions or greater levels of concern. An independent samples t-test was used for comparisons between two groups, while one-way analysis of variance (ANOVA) was applied for comparisons across multiple groups. Where assumptions of normality were not met, non-parametric tests (Mann–Whitney U test or Kruskal–Wallis test) were used. A *p*-value < 0.05 was considered statistically significant.

## Results

A total of 400 participants were approached to take part in the study; however, only 340 completed questionnaires were returned and included in the final analysis, yielding a response rate of 85%. The stated reasons for declining the survey were either a lack of time or interest. The majority of participants were females (62%) compared to males (38%). The mean ± SD age was 37.8 ± 6.47. The demographic characteristics of the recruited participants were displayed in Table 1.

**Table 1:** Demographic characteristics of study participants (*n* = 340).

Variable	Category/Measurement	Frequency (n, %)
Age (years)	Mean ± SD	37.8 ± 6.47
Gender	Female	211 (62%)
	Male	129 (38%)
Education	Bachelor	256 (75%)
	Master	76 (22%)
	PhD	8 (3%)
Type of Pharmacy	Community Pharmacy	228 (67%)
	Hospital Pharmacy	112 (33%)
Years of Experience	1–5 years	197 (58%)
	6–10 years	102 (30%)
	>10 years	41 (12%)
Nationality	Egyptian	79 (23%)
	Jordanian	56 (16%)
	Syrian	35 (10%)
	Sudanese	20 (6%)
	Indian	113 (33%)
	Emirati	12 (4%)
	Other	25 (7%)
Average daily visitors	≤10	31 (9%)
	11–50	187 (55%)
	>50	122 (36%)
Basic AI understanding	Strongly agree	51 (15%)
	Agree	61 (18%)
	Neutral	71 (21%)
	Disagree	68 (20%)
	Strongly disagree	89 (26%)

The largest group of participants was Indians (33.2%), Egyptians (23.2%), Jordanians (16.4%), Syrians (10.4%), Sudanese (5.9%), Emiratis (3.6%), and others (7.3%). Most respondents worked as community pharmacists (65.5%) or hospital Pharmacists (34.5%). The Educational level varied among participants, with a significant number holding a bachelor's degree in pharmacy (75.3%).

The level of basic AI understanding varied among participants. A small percentage of participants strongly agreed/agreed that they have basic AI understanding (33%), compared to 46% who disagreed/strongly disagreed with the statement, and 21% who were neutral about AI understanding.

As shown in Table 2, upon investigating pharmacists' concerns and barriers towards the implementation of AI in pharmacy practice, the majority of participants expressed concerns that AI could replace pharmacists in the foreseeable future (3.9 ± 1.1), make healthcare professionals expendable ((4.0 ± 1.0), and make the pharmacy career less attractive to pursue (3.5 ± 1.2).

**Table 2:** Mean scores of participants' perceived concerns and barriers towards AI implementation in pharmacy practice (*n* = 340).

No	Statement	Mean ± SD
1	AI will replace general pharmacists	3.9 ± 1.1
2	AI will make healthcare professionals expendable	4.0 ± 1.0
3	AI reduces the attractiveness of pharmacy careers	3.5 ± 1.2
4	AI devalues the medical profession	3.6 ± 1.1
5	AI negatively affects pharmacist–patient trust	3.5 ± 1.1
6	AI is vulnerable to cybersecurity threats	4.2 ± 0.8
7	AI poses a risk to patient data privacy	4.1 ± 0.9
8	AI may produce biased responses	3.9 ± 1.0
9	AI may generate inaccurate responses	3.9 ± 1.0
10	High cost limits AI accessibility	4.0 ± 1.0
11	Limited access to AI technologies	3.8 ± 1.1
12	Lack of legal regulation	3.3 ± 1.1
13	Lack of standardization across systems	3.7 ± 1.0
14	Lack of pharmacist training	3.2 ± 1.2
15	Patients are apprehensive about AI decisions	3.8 ± 1.0
16	Physicians are apprehensive about AI decisions	3.7 ± 1.0
17	AI reduces human interaction/empathy	4.0 ± 0.9
18	AI may oversell unnecessary products	3.4 ± 1.2

Participants expressed considerable concerns regarding patient data privacy (4.1 ± 0.9) and the vulnerability of AI systems to cybersecurity threats, including hacking and data breaches (4.2 ± 0.8). Additional challenges included the high cost of AI implementation (4.0 ± 1.0) and the limited accessibility of AI technologies within healthcare systems (3.8 ± 1.1). A high level of concern was also reported regarding the potential dehumanization of the profession due to reduced communication and empathy (4.0 ± 0.9). Furthermore, participants indicated that both patients (3.8 ± 1.0) and physicians (3.7 ± 1.0) may be apprehensive about AI's ability to develop appropriate treatment plans. Moderate concern was observed regarding the potential for AI systems to promote unnecessary products (3.4 ± 1.2). In contrast, lower concern levels were reported for lack of pharmacist training (3.2 ± 1.2) and absence of comprehensive legal regulation (3.3 ± 1.1). Additionally, participants expressed concern that AI integration may devalue the medical profession (3.6 ± 1.1) and highlighted the lack of standardized AI systems across different practice settings as an important barrier (3.7 ± 1.0). Many participants also expressed concerns regarding the potential for AI to generate inaccurate or biased responses (3.9 ± 1.0) or replace a general pharmacist (3.9 ± 1.1).

As shown in Table 3, pharmacists demonstrated generally positive perceptions regarding the benefits of AI in pharmacy practice. The highest mean scores were observed for AI's ability to multitask and analyse data efficiently (4.1 ± 0.9), improve pharmacy services (3.9 ± 1.0), and enhance the pharmacist's role in patient follow-up (3.8 ± 1.1). In contrast, more moderate perceptions were reported for improving patient satisfaction (3.5 ± 1.1), reducing medication errors (3.3 ± 1.2), and enhancing tailored pharmaceutical care plans (3.6 ± 1.0). Lower mean scores were observed for AI's ability to assist in clinical investigation tasks (3.2 ± 1.2), improve patient outcomes (3.5 ± 1.1), and reduce the cost of care (3.1 ± 1.2).

**Table 3:** Mean scores of participants perceived benefits of AI implementation in pharmacy practice (*n* = 340) .

No.	Statement	Mean ± SD
1	AI improves pharmacy services	3.9 ± 1.0
2	AI improves patient satisfaction	3.5 ± 1.1
3	AI reduces medication errors	3.3 ± 1.2
4	AI enhances tailored care plans	3.6 ± 1.0
5	AI improves clinical investigation tasks	3.2 ± 1.2
6	AI enhances patient follow-up	3.8 ± 1.1
7	AI can multitask and analyze data quickly	4.1 ± 0.9
8	AI improves patient outcomes	3.5 ± 1.1
9	AI reduces the cost of care	3.1 ± 1.2

As shown in Table 4, several demographic factors, including basic AI understanding, years of experience, daily patient volume, and type of pharmacy, significantly influenced pharmacists' perceptions of AI. Pharmacists with higher levels of AI understanding demonstrated more positive perceptions and fewer concerns compared to those with lower levels of knowledge (*p* < 0.05), suggesting that familiarity with AI enhances acceptance.

**Table 4:** Association between participants' demographic data and their perception and concerns regarding AI implementation in practice (*n* = 340).

Variable	Category	Benefit Score (Mean ± SD)	*p*-value	Concern Score (Mean ± SD)	*p*-value
Gender	Female	3.7 ± 0.6	0.265	3.9 ± 0.7	0.148
	Male	3.8 ± 0.6		3.8 ± 0.7	
Education	Bachelor	3.7 ± 0.6	0.123	3.9 ± 0.7	0.081
	Master	3.8 ± 0.6		3.8 ± 0.7	
	PhD	3.9 ± 0.5		3.7 ± 0.6	
Type of Pharmacy	Community	3.6 ± 0.6	0.506	4.0 ± 0.7	0.042^*^
	Hospital	3.8 ± 0.5		3.7 ± 0.7	
Years of Experience	1–5 yrs	3.8 ± 0.6	0.031^*^	4.0 ± 0.7	0.021^*^
	6–10 yrs	3.7 ± 0.6		3.8 ± 0.7	
	>10 yrs	3.5 ± 0.6		3.6 ± 0.7	
Nationality	Egyptian	3.7 ± 0.6	0.252	3.9 ± 0.7	0.174
	Jordanian	3.7 ± 0.6		3.8 ± 0.7	
	Syrian	3.7 ± 0.6		3.8 ± 0.7	
	Sudanese	3.6 ± 0.6		3.9 ± 0.7	
	Indian	3.8 ± 0.6		3.8 ± 0.7	
	Emirati	3.8 ± 0.5		3.7 ± 0.6	
	Other	3.7 ± 0.6		3.9 ± 0.7	
Daily Visitors	≤10	3.5 ± 0.7	0.012^*^	4.1 ± 0.7	0.005^*^
	11–50	3.7 ± 0.6		3.9 ± 0.7	
	>50	3.9 ± 0.5		3.7 ± 0.7	
Basic AI Understanding	Strongly agree	4.0 ± 0.5	0.035^*^	3.5 ± 0.6	0.002^*^
	Agree	3.8 ± 0.6		3.8 ± 0.7	
	Neutral	3.7 ± 0.6		3.9 ± 0.7	
	Disagree	3.5 ± 0.7		4.1 ± 0.7	
	Strongly Disagree	3.4 ± 0.7		4.2 ± 0.6	

* indicates statistically significant difference (*p* < 0.05).

Similarly, pharmacists with fewer years of experience and those working in high-volume settings (>50 daily visitors) reported more positive perceptions toward AI implementation (*p* < 0.05), which may reflect greater openness to technological innovation and the perceived usefulness of AI in managing workload demands.

In contrast, community pharmacists reported significantly higher levels of concern compared to hospital pharmacists (*p* = 0.042), possibly due to differences in practice environment, infrastructure, and exposure to advanced healthcare technologies.

Significant differences in perception scores were observed across several demographic variables. Pharmacists with higher levels of AI understanding demonstrated higher perceived benefit scores and lower concern scores (*p* < 0.05), indicating that familiarity with AI is associated with greater acceptance.

Additionally, pharmacists with fewer years of experience reported more positive perceptions compared to those with longer experience (*p* < 0.05), suggesting that early-career professionals may be more receptive to technological innovation.

Pharmacists working in high-volume settings also demonstrated higher benefit scores and lower concern scores (*p* < 0.05), which may reflect the perceived usefulness of AI in managing increased workload. Furthermore, community pharmacists reported significantly higher concern scores compared to hospital pharmacists (*p* = 0.042), highlighting differences in perception which may be based on practice setting.

## Discussion

Artificial intelligence (AI) is a promising advancement that could revolutionise every aspect of healthcare, including diagnosis, disease management, and improved patient treatment outcomes (17). However, its implementation still continues to raise important challenges and concerns that must first be addressed (18). This study explored pharmacists' perceptions of the benefits and barriers associated with AI implementation in pharmacy practice, with findings that may help inform targeted strategies to support its effective integration.

A substantial proportion of pharmacists perceive that AI could improve pharmacy services, enhance multitasking capabilities, and support more efficient patient follow-up. These findings are consistent with previous studies demonstrating that AI can process large volumes of data and support personalized medication management by identifying drug interactions, contraindications, adverse effects, and prescription errors (19–21).

However, pharmacists' perceptions of AI's clinical impact were more cautious. Fewer pharmacists believed that AI could improve patient outcomes, reduce medication errors, or lower healthcare costs, suggesting a gap between perceived operational benefits and confidence in its clinical applicability. While previous studies have demonstrated the ability of AI to analyse large-scale datasets and support clinical decision-making (22, 23), much of this evidence is derived from controlled or retrospective settings, limiting its applicability in routine clinical practice. For instance, studies have shown that AI can analyse large-scale electronic health records (EHRs) and predict treatment responses, such as antidepressant outcomes (24, 25). Nevertheless, other studies have reported limited confidence in AI's ability to reliably analyse patient data and deliver personalized treatment recommendations (26).

Furthermore, the potential applications of AI in pharmacy practice have been expanded with the emergence of generative artificial intelligence (GenAI). These advanced systems can support a wide range of tasks, including patient counselling, clinical documentation, drug information retrieval, and health education, thereby enhancing workflow efficiency and supporting a more patient-centred approach (27, 28). However, despite these promising applications, the use of GenAI introduces important challenges, including concerns regarding the accuracy and reliability of AI-generated outputs, particularly the risk of misleading or fabricated information, as well as ethical considerations related to accountability, data privacy, and confidentiality (29). Emerging evidence further highlights that gaps in AI literacy among healthcare professionals may compromise safe use and patient outcomes, underscoring the need for cautious and well-regulated integration of GenAI technologies into healthcare systems (30, 31).

IIn addition, several studies have highlighted the capability of AI to predict hospitalization and mortality (22), anticipate patient deterioration (23), support decision-making in sepsis management (32), and derive treatment policies from observational data (33).

These findings may be particularly relevant to the integration of AI into core pharmacy services, such as medication therapy management (MTM). This aims at optimizing medication use through review, counselling, and follow-up; however, its implementation remains inconsistent due to limited training and lack of collaboration. Therefore, AI may support MTM by enhancing data analysis and clinical decision-making, although concerns regarding its reliability and real-world applicability may influence its adoption (34).

In addition, AI systems may face challenges related to data fragmentation, limited interoperability, and potential biases, which can affect their performance and generalizability in real-world settings (35–37). These factors may contribute to the cautious perceptions observed among pharmacists regarding AI's clinical effectiveness.

Participants also expressed concerns regarding patient and physician acceptance of AI-assisted decision-making. This is consistent with previous research indicating that while individuals may accept AI for general health-related tasks, they remain hesitant to rely on it for critical clinical decisions (20, 38). Trust in AI may vary based on demographic and experiential factors, including familiarity with technology and prior exposure (39, 40).

Economic and infrastructural barriers were also prominent. The high cost of AI implementation, including infrastructure, software, and training requirements, was perceived as a major limitation. Additionally, concerns related to data privacy and cybersecurity were frequently reported, consistent with existing literature emphasizing the importance of robust data protection frameworks (41, 42).

Concerns regarding the potential impact of AI on pharmacists' roles were evident, with previous studies documenting apprehension about job displacement (43). While AI adoption in healthcare continues to expand, current evidence suggests that it is more likely to transform rather than replace pharmacists' roles, by shifting responsibilities toward oversight and integration of AI-driven systems (44). Nevertheless, concerns related to job security and the need to acquire new competencies may influence pharmacists' acceptance and readiness to adopt AI technologies.

The lack of standardized AI systems and comprehensive regulatory frameworks was another key concern. Effective integration of AI into pharmacy practice requires clear regulatory guidelines, standardization across healthcare systems, and collaboration between healthcare professionals and technology developers (37).

Finally, the findings of this study may help inform healthcare policymakers by providing insights into pharmacists' perspectives on AI. Understanding these perceptions is essential for guiding future implementation strategies and ensuring that AI is integrated in a manner that supports, rather than compromises, patient care.

### Limitations

This study has several limitations that should be considered when interpreting the findings. First, the use of a cross-sectional design limits the ability to establish causal relationships and reflects pharmacists' perceptions at a single point in time. Second, convenience sampling and recruitment through professional networks may introduce selection bias and limit representativeness. Despite the high response rate, non-response bias cannot be excluded, as non-participants may differ from respondents in their perceptions or experiences with artificial intelligence. These factors may limit generalizability to the broader pharmacist population in the UAE. Third, data were collected using a self-administered questionnaire, which may be subject to response bias, including social desirability and recall bias. Although the questionnaire demonstrated excellent internal consistency based on Cronbach's alpha values, additional psychometric analyses, such as exploratory factor analysis, were not conducted, which may limit the robustness of construct validity. Fourth, the absence of multivariate analysis limits the ability to identify independent predictors while controlling for potential confounding variables. Finally, the study was conducted in the UAE, and therefore, its findings may not be generalizable to pharmacists in other countries with different healthcare systems, regulatory frameworks, and levels of AI adoption.

Despite its limitations, this study provides valuable insights into pharmacists' perceptions of the benefits and concerns of artificial intelligence (AI) in the United Arab Emirates, where evidence remains limited. It contributes to the existing literature by highlighting the gap between the perceived AI operational benefits and concerns regarding clinical applicability, ethical implications, and professional roles.

Future research should build on these findings by adopting multivariate analyses to identify independent predictors of AI acceptance. Additionally, further studies are needed to evaluate the real-world impact of AI integration on clinical outcomes and core pharmacy services.

## Conclusion

This study demonstrated that pharmacists generally hold positive perceptions toward the use of artificial intelligence (AI) in pharmacy practice, alongside important concerns related to data privacy, accuracy, and regulatory issues. These findings highlight a gap between perceived benefits, such as improved efficiency and patient care, and concerns regarding clinical reliability and implementation challenges. Addressing this gap requires strengthening pharmacists' preparedness through updated pharmacy education, targeted training, and the development of clear regulatory frameworks to support the safe and effective integration of AI into practice. Further large-scale studies are needed to address key unanswered questions, including whether AI can truly improve patient outcomes, reduce costs, and be effectively regulated in clinical practice.

## Data Availability

The raw data supporting the conclusions of this article will be made available by the authors, without undue reservation.
